# Confounding Factors in the Diagnosis of Hereditary Spherocytosis and Gallstone Formation in Related Hemolytic Disorders From a Tertiary Care Center in North India

**DOI:** 10.7759/cureus.96168

**Published:** 2025-11-05

**Authors:** Rizwan Athar, Rajesh Kashyap, Jalaj Gupta

**Affiliations:** 1 Hematology, Sanjay Gandhi Postgraduate Institute of Medical Sciences, Lucknow, IND

**Keywords:** beta-thalassemia, bilirubin, g6pd deficiency, hemolysis, ultrasonography

## Abstract

Introduction: Gallstones are a significant complication in individuals with hereditary spherocytosis (HS) and related hemolytic disorders. Chronic hemolysis leads to elevated bilirubin levels, a precursor for pigment gallstone formation. Despite advancements in understanding HS, the mechanisms driving gallstone pathogenesis remain incompletely understood, particularly in the presence of genetic factors such as Gilbert syndrome, beta-thalassemia trait, and G6PD deficiency.

Objective: This study aims to investigate gallstone patterns in individuals with HS and related hemolytic conditions, focusing on the role of genetic, metabolic, and clinical factors in gallstone development.

Materials and methods: A five-year prospective observational study was conducted on 100 patients with HS and related conditions. Clinical, hematological, biochemical, and genetic data were analyzed. Gallstone presence was confirmed via ultrasonography. Correlations between gallstone incidence and various parameters, including bilirubin levels, hemoglobin (Hb) levels, spleen size, and genetic predisposition, were assessed. Statistical significance was determined using chi-square correlation and regression analyses.

Results: Gallstones were present in 42% of the cohort, with increased incidence in participants with homozygous Gilbert syndrome (28%) compared to heterozygous individuals (20%; p = 0.04). Hb levels <12.5 g/dL were significantly associated with gallstone presence (71%; p < 0.05). Elevated total bilirubin correlated positively with gallstones (r = +0.63; p < 0.001), while increased spleen size showed a strong negative correlation (ρ = -0.744; p < 0.001).

Conclusions: The development of gallstones in hemolytic disorders involves a multifactorial interplay of hemolysis, bilirubin metabolism, and genetic factors. Homozygous Gilbert syndrome significantly increases the susceptibility to gallstones, highlighting the importance of genetic screening in at-risk patients.

## Introduction

Gallstones are a common clinical complication arising in individuals with hereditary spherocytosis (HS) and related hemolytic disorders [1-3]. HS is a hereditary condition characterized by abnormally shaped red blood cells (spherocytes) prone to hemolysis [4-5]. This chronic hemolysis results in an increased release of bilirubin, a byproduct of heme catabolism, which plays a crucial role in the formation of pigment gallstones. The coexistence of gallstone disease with HS and related hemolytic disorders like thalassemia and glucose-6-phosphate dehydrogenase (G6PD) deficiency represents a rare but clinically significant phenomenon. The rarity of this co-occurrence underscores the complex interplay of genetic, metabolic, and environmental factors that govern gallstone pathogenesis. Despite advances in the understanding of HS and its management, the mechanisms underlying gallstone development in these patients remain incompletely elucidated [6-7]. Recent studies have highlighted the role of genetic variability in gallstone susceptibility among individuals with hereditary hemolytic conditions [8-11]. For instance, variations in genes associated with bilirubin metabolism, such as UGT1A1, may exacerbate the risk of gallstone formation [12-13]. This study investigates the patterns of gallstone development in individuals with HS and related hemolytic disorders, aiming to delineate the contributory factors, temporal trends, and clinical outcomes associated with gallstone formation. By examining these patterns, we aim to deepen our understanding of gallstone pathophysiology in hemolytic conditions and contribute to the development of more personalized management strategies for patients affected by this condition.

## Materials and methods

Study design

This prospective observational study was conducted in the Department of Hematology at the Sanjay Gandhi Postgraduate Institute of Medical Sciences, Lucknow, from June 2020 to May 2025. The objective was to evaluate the patterns and incidence of gallstone formation among patients with HS and related hemolytic disorders.

Patient recruitment

All newly diagnosed patients with HS and related hemolytic disorders, such as thalassemia and G6PD, presenting to the hematology OPD were included in the study. Family history was also taken. Patients with liver and renal diseases were excluded from the study. Secondary causes of hemolytic anemia, like inflammatory conditions, paroxysmal nocturnal hemoglobinuria (PNH), and autoimmune hemolytic anemia, were also excluded from the study. The Institutional Ethics Committee of the Sanjay Gandhi Postgraduate Institute of Medical Sciences issued approval 2023-163-DM-133, and consent was obtained from the patients and their guardians for the prospective investigations.

Data collection

A total of 100 patients were enrolled, who presented with anemia, splenomegaly, and jaundice. The diagnosis relied on patient history, clinical signs, and laboratory tests, including blood smears showing spherocytes, reticulocyte counts, and specific tests such as a flow cytometry-based osmotic fragility test and an eosin-5′-maleimide binding test. Demographic details, clinical characteristics, and laboratory data were systematically collected. Demographic parameters included age and sex, while hematological indices comprised hemoglobin (Hb), lactate dehydrogenase (LDH), mean corpuscular volume (MCV), mean corpuscular hemoglobin (MCH), and MCH concentration. Biochemical parameters recorded included total bilirubin and unconjugated bilirubin. Splenic assessment was performed through measurement of splenic size and evaluation of splenic status. Specialized diagnostic evaluations were conducted to confirm the presence of hemolytic disorders.

High-performance liquid chromatography was utilized for Hb variant analysis. Genetic testing for Gilbert syndrome was performed by analyzing mutations in the UGT1A1 gene. Additional enzyme assays were performed for G6PD deficiency and PNH. Reticulocyte counts and direct and indirect Coombs tests were also performed to support diagnostic classification.

Gallstone assessment was carried out using ultrasonography. The presence or absence of gallstones was documented, and patients were categorized according to their underlying hemolytic condition.

Statistical analysis

Data were analyzed using SPSS Statistics version 28.0 (IBM Corp., Released 2021. IBM SPSS Statistics for Windows, Version 28.0. Armonk, NY: IBM Corp.). Continuous variables were expressed as mean ± standard deviation, while categorical variables were summarized as frequencies and percentages. Comparisons between categorical variables were performed using the chi-square test or Fisher’s exact test, as appropriate. Correlations between clinical or laboratory parameters and gallstone incidence were assessed using Spearman’s correlation coefficients. A p-value < 0.05 was considered statistically significant.

## Results

Demographic characteristics

The study population (n = 100) had a mean age of 25.7 ± 11.7 years. The majority of patients were between 11 and 30 years old (n = 61, 61%). Age distribution was as follows: 0-10 years (n = 6, 6%), 11-20 years (n = 30, 30%), 21-30 years (n = 31, 31%), 31-40 years (n = 19, 19%), 41-50 years (n = 12, 12%), and 51-60 years (n = 2, 2%) (Table 1). The sex distribution was nearly equal, with 48 males (48%) and 52 females (52%).

**Table 1:** Distribution of age groups among study participants Values are presented as a percentage of the total cohort (n = 100). SD: standard deviation

Age group	Percentage (N = 100)
0-10	6%
11-20	30%
21-30	31%
31-40	19%
41-50	12%
51-60	2%
Mean ± SD	25.7 ± 11.7 years

Hematological and biochemical parameters

**Figure 1:**
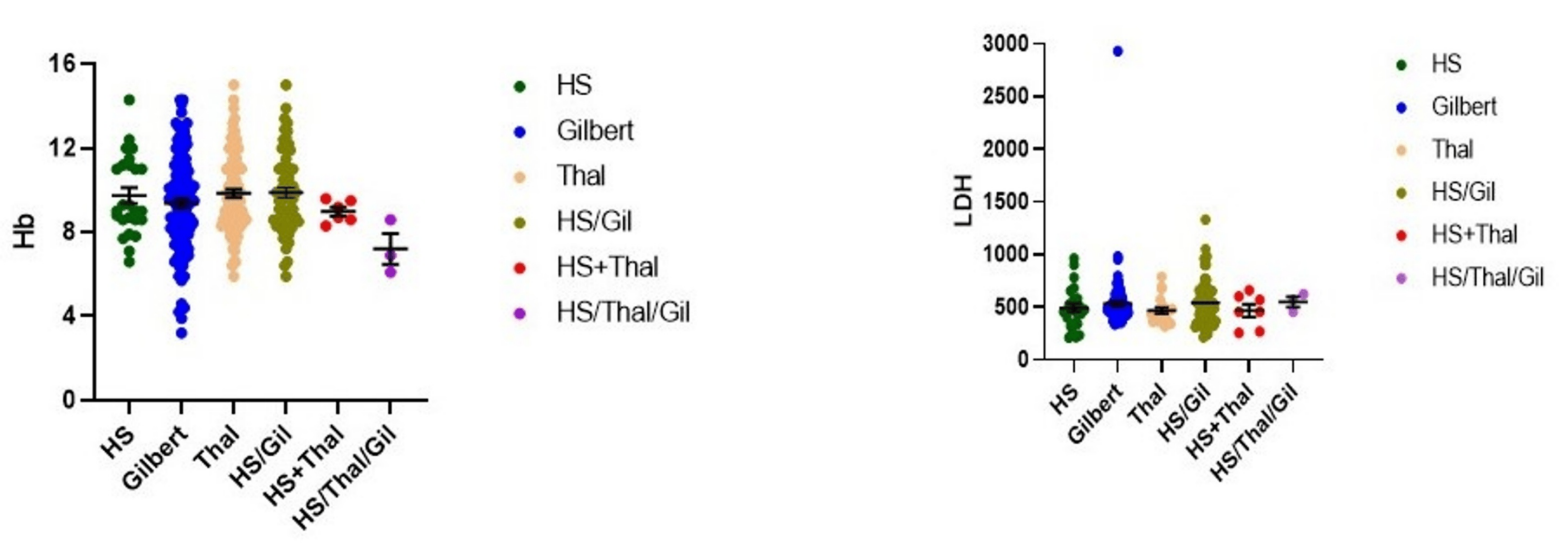
Hb levels and LDH level among study participants Elevated LDH values indicate increased hemolysis, while lower Hb levels correspond to higher gallstone incidence. Hb: hemoglobin, LDH: lactate dehydrogenase

Hb levels (mean 12.22 ± 0.46 g/dL) showed the following distribution: <11 g/dL (n = 69, 69%), 11-12.5 g/dL (n = 24, 24%), and >12.5 g/dL (n = 7, 7%) (Table 2). LDH levels were elevated in most participants: ≥600 U/L (n = 71, 71%) and <600 U/L (n = 29, 29%) (Table 3). Elevated LDH values indicate increased hemolysis, while lower Hb levels correspond to a higher incidence of gallstones (Figure 1).

**Table 2:** Distribution of Hb levels among study participants (n = 100) SD: standard deviation, Hb: hemoglobin

Range	Percentage (N = 100)
<11 g/dl	69%
11-12.5 g/dl	24%
>12.5 g/dl	7%
Mean ± SD	12.22 ± 0.46 g/dl

**Table 3:** Distribution of LDH levels among study participants (n = 100) LDH levels were categorized into two groups: <600 U/L and >600 U/L. LDH: lactate dehydrogenase

Range	Percentage (N = 100)
≥600	71%
<600	29%

Gallstone incidence

**Figure 2:**
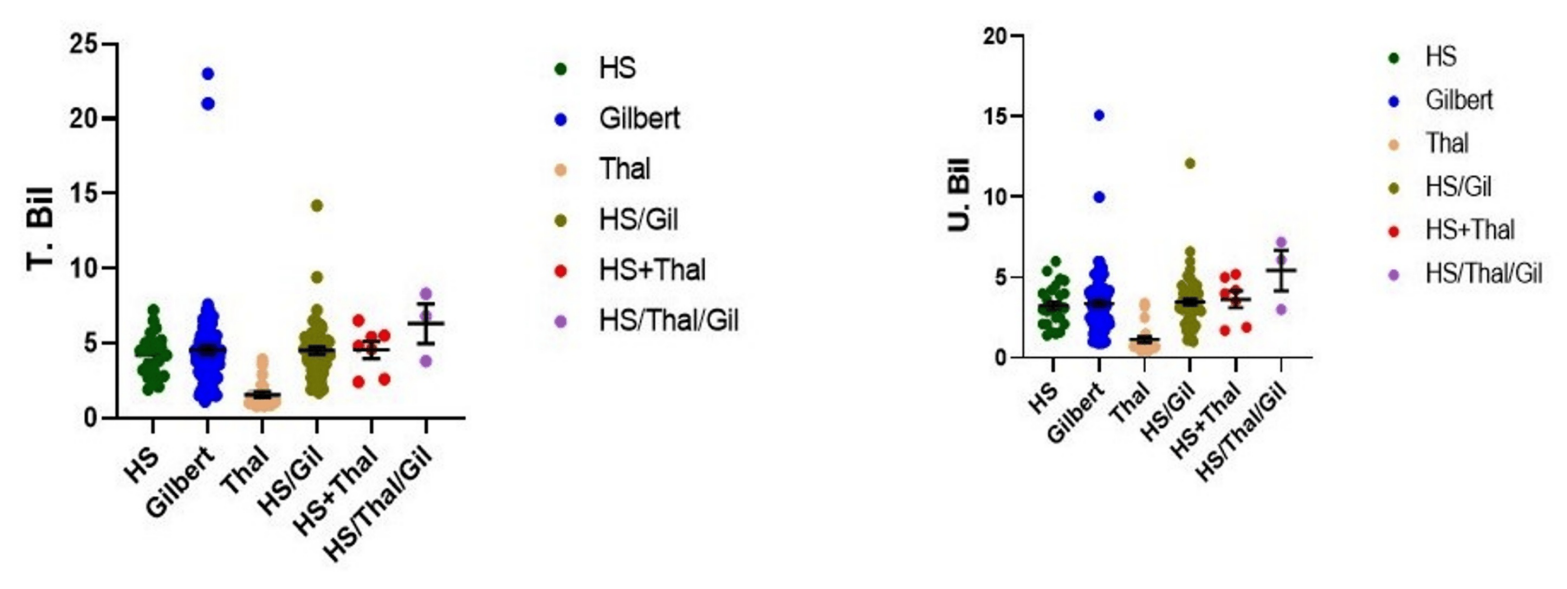
Total bilirubin and unconjugated bilirubin levels among study participants Higher total and unconjugated bilirubin levels were significantly associated with gallstone presence, emphasizing the role of bilirubin metabolism in gallstone pathogenesis.

Gallstones were detected in 42 patients (42%), while 58 patients (58%) had no gallstones. A relationship was noted between Hb concentration and gallstone status. Among those with Hb <11 g/dL, 19 patients (28%) had gallstones and 50 (72%) did not. In the 11-12.5 g/dL group, 10 (42%) had gallstones, while 14 (58%) did not. In patients with Hb > 12.5 g/dL, two (29%) had gallstones, while five (71%) did not. The association was statistically significant in the highest Hb group (p<0.05) (Table 4). Higher total and unconjugated bilirubin levels were significantly associated with cholelithiasis (Figure 2).

**Table 4:** Association between Hb levels and gallstone status among study participants Participants were stratified into Hb categories, and the incidence of gallstones was compared between groups. Hb: hemoglobin

Hb range	Gallstone present (%)	Gallstone absent (%)	p-value
<11 g/dL (n = 69)	19 (28%)	50 (72%)	0.12
11-12.5 g/dL (n = 24)	10 (42%)	14 (58%)	0.08
>12.5 g/dL (n = 7)	2 (29%)	5 (71%)	<0.05

Genetic associations

Gilbert syndrome genotyping revealed that 28 patients (28%) were homozygous, 40 (40%) were heterozygous, and 32 (32%) were negative. Gallstones were present in 21 of 28 homozygous patients (75%), 20 of 40 heterozygous patients (50%), and none of the 32 negative patients (0%). This association was statistically significant (p = 0.04) (Table 5). Other genetic traits did not show significant associations. Gallstones occurred in two of six patients (33%) with beta-thalassemia trait and in two of six patients (33%) with G6PD deficiency (p = 0.68 for both) (Table 6).

**Table 5:** Distribution of Gilbert syndrome status and gallstone presence among participants (n = 68) Genotyping results of Gilbert syndrome are shown as homozygous and heterozygous.

Gilbert syndrome status	Gallstone present	Gallstone absent	Total (n = 68)
Homozygous	21 (75%)	7 (25%)	28 (100%)
Heterozygous	20 (50%)	20 (50%)	40 (100%)

**Table 6:** Association between genetic traits and gallstone status among study participants The presence of gallstones was analyzed in relation to Gilbert syndrome heterozygosity, beta-thalassemia trait, and G6PD deficiency. G6PD: glucose-6-phosphate dehydrogenase

Genetic trait	Gallstone present (%)	Gallstone absent (%)	p-value
Gilbert syndrome heterozygous (n = 40)	18 (45%)	22 (55%)	0.04
Beta-thalassemia trait (n = 6)	2 (33%)	4 (67%)	0.68
G6PD positive (n = 6)	2 (33%)	4 (67%)	0.68

Correlation analysis

Correlation analysis revealed a moderate positive correlation between gallstone incidence and Hb concentration (r = 0.528, p = 1.69 × 10⁻⁸) (Table 7). Spleen size showed a strong negative correlation with gallstone presence (p = -0.744, p = 7.8×10⁻¹⁹) (Table 7). Biochemical correlations further emphasized the role of bilirubin metabolism. Gallstone incidence correlated with total bilirubin (r = +0.63, p < 0.001) and unconjugated bilirubin (r = +0.42, p = 0.01). Hb levels correlated negatively with gallstones (r = -0.35, p = 0.02) (Table 8). A chi-square test confirmed the significance (χ² = 8.85, p = 0.0029) (Table 9).

**Table 7:** Correlation analysis of gallstone presence with clinical parameters Correlation coefficients (Pearson or Spearman as appropriate) are provided for Hb and spleen size in relation to gallstone incidence. Hb: hemoglobin

Correlation pair	Coefficient	p-value	Interpretation
Gallstone vs. Hb	Г = 0.528	1.69×10^-8^	Moderate positive correlation
Gallstone vs. spleen size	ρ = -0.744	7.8×10^-19^	Strong negative correlation

**Table 8:** Correlation analysis of gallstone presence with biochemical parameters Hb: hemoglobin

Parameter	Correlation coefficient (r)	p-value
Total bilirubin	+0.63	<0.001
Unconjugated bilirubin	+0.42	0.01
Hb	–0.35	0.02

**Table 9:** Chi-square analysis of Gilbert syndrome and gallstone status

Chi-square comparison	Chi-square vale	p-value	Interpretation
Gallstone vs. Gilbert syndrome	8.85	0.0029	Significant (p < 0.05)

## Discussion

The findings of this study provide valuable insights into the complex interplay between hemolytic disorders, genetic factors, and gallstone formation, particularly in individuals with HS and related conditions. Gallstones, a common complication of chronic hemolysis, were present in 42% of the study population, suggesting their clinical relevance in these patients.

The results reinforce the established link between hemolysis and the development of gallstones. Chronic hemolysis in conditions such as HS leads to increased bilirubin production, a critical substrate for pigment gallstone formation. This is supported by the significant positive correlation between total bilirubin levels and gallstone incidence (r = +0.63, p < 0.001). Elevated unconjugated bilirubin levels also showed a notable association with gallstones (r = +0.42, p = 0.01), further highlighting the role of bilirubin metabolism in gallstone pathogenesis. However, LDH, a nonspecific marker of hemolysis, did not show a significant correlation with gallstone presence (p = 0.15), suggesting that its role as a predictor of gallstone risk may be limited in this context.

Genetic predisposition emerged as a significant factor influencing gallstone development. Homozygous Gilbert syndrome was strongly associated with an increased risk of gallstones (p = 0.04). Gilbert syndrome is characterized by reduced activity of the UGT1A1 enzyme, which impairs bilirubin conjugation and leads to its accumulation. This finding aligns with previous research suggesting that genetic variations in bilirubin metabolism pathways exacerbate gallstone susceptibility in hemolytic disorders. In contrast, no significant associations were observed for thalassemia or G6PD status, suggesting that these genetic traits may not substantially contribute to gallstone pathogenesis in this cohort.

Splenomegaly was observed in 54% of participants and demonstrated a strong negative correlation with gallstone presence (p = -0.744, p = 7.8 × 10⁻¹⁹). This inverse relationship may reflect the role of splenectomy, a common treatment for HSP, in increasing the risk of gallstones. Following splenectomy, the reduced clearance of bilirubin and spherocytes may contribute to the formation of gallstones. This finding supports the need for vigilant monitoring of gallstone risk in patients undergoing splenectomy.

The study also highlighted an inverse relationship between Hb levels and the presence of gallstones (r = -0.35, p = 0.02). Participants with Hb levels <12.5 g/dL were significantly more likely to develop gallstones (p < 0.05). This association reflects the exacerbation of hemolysis in severe anemia, leading to increased bilirubin production. The findings emphasize the importance of Hb monitoring in patients with HS and related disorders.

Clinical implications and future directions

These results emphasize the multifactorial nature of gallstone development in hemolytic conditions, driven by the interplay of hemolysis, bilirubin metabolism, and genetic predisposition. Clinicians should consider comprehensive screening for gallstones in patients with HS, particularly those with Gilbert syndrome or severe anemia [14]. The findings suggest that targeting bilirubin metabolism pathways may represent a therapeutic avenue for mitigating gallstone risk.

Future research should focus on longitudinal studies to elucidate the temporal dynamics of gallstone formation and the impact of specific interventions, such as splenectomy or pharmacological modulation of bilirubin metabolism. Genetic studies exploring polymorphisms in UGT1A1 and related genes could further clarify their role in gallstone susceptibility, paving the way for personalized management strategies [15].

Limitations of the study

The sample size, while reasonable, requires expansion for broader validation and inclusion in the diagnostic protocol for Indian patients. The findings, although statistically significant, require further validation in large, diverse cohorts. The diagnosis of HS could not be confirmed by polyacrylamide gel electrophoresis or genetic testing.

## Conclusions

This research sheds light on the complex mechanisms driving gallstone formation in individuals with HS and related hemolytic disorders. By identifying significant correlations between gallstone presence and factors such as bilirubin metabolism, Hb levels, spleen size, and genetic predisposition, the study highlights the intricate interplay of metabolic and genetic factors in gallstone pathogenesis among these patients.

A key contribution of this research is the discovery of a notable association between homozygous Gilbert syndrome and gallstone susceptibility, underscoring the role of genetic variability in bilirubin metabolism as a key determinant of gallstone risk. The inverse relationship between spleen size and gallstone formation provides new insights into the potential long-term effects of splenectomy on gallstone development.

This study enhances our understanding of gallstone pathophysiology in hemolytic disorders. It emphasizes the value of integrating genetic screening and personalized monitoring into the clinical management of patients with HS. These findings lay the groundwork for future research into targeted therapeutic interventions to mitigate the risk of gallstones in this vulnerable population.
